# Supplementary material for: Associations among Alcohol Drinking, Smoking, and Nonrestorative Sleep: A Population-Based Study in Japan
Source: Clocks Sleep. 2022 Oct 24;4(4):595–606. doi: 10.3390/clockssleep4040046 (PMC9680481; doi:10.3390/clockssleep4040046)
Supplement: Supplementary file 1 [file clockssleep-04-00046-s001.zip › clockssleep-1905533-supplementary.pdf]

Table S1. Prevalence of NRS among non-SSD individuals by demographic characteristics

		Men (N= 23,459)			Women (N=24,506)		
		%	95%CI	p-value	%	95%CI	p-value
Total		10.9	10.5 - 11.3		10.3	9.9 - 10.6	
Age class							
	20-29	12.4	11.1 - 13.7	<0.001	11.9	10.7 - 13.2	<0.001
	30-39	16.6	15.4 - 17.9		14.3	13.2 - 15.4	
	40-49	15.2	14.0 - 16.4		15.3	14.1 - 16.5	
	50-59	13.1	12.0 - 14.3		12.6	11.5 - 13.8	
	60-69	7.5	6.8 - 8.3		6.7	6.0 - 7.4	
	70-79	5.5	4.8 - 6.3		5.6	4.9 - 6.4	
	80+	5.7	4.7 - 7.0		6.7	5.8 - 7.7	
Alcohol/day							
	None	11.2	10.6 - 11.9	<0.001	9.8	9.4 - 10.3	<0.001
	≥0, <23g	9.9	9.2 - 10.6		10.6	9.7 - 11.5	
	≥23, <46g	10.4	9.5 - 11.3		12.8	10.9 - 14.9	
	≥46, <69g (men) ≥46g (women)	11.4	10.0 - 12.9		16.3	13.3 - 19.7	
	≥69g (men)	16.7	14.5 - 19.1		-	- - -	
Smoking							
	Nonsmoker	9.9	9.5 - 10.4	<0.001	9.9	9.5 - 10.3	<0.001
	Light smoker	12.5	11.7 - 13.4		14.6	13.1 - 16.2	
	Heavy smoker	15.2	13.4 - 17.2		12.9	8.6 - 18.4	

Mental distress										
Light	7.1	6.7	-	7.5	<0.001	6.3	5.9	-	6.6	<0.001
Moderate	20.8	19.6	-	22.0		19.4	18.4	-	20.5	
Serious	41.7	37.8	-	45.7		32.9	29.7	-	36.3	

Participants for whom data were missing were excluded from the analyses.

Abbreviations: NRS: nonrestorative sleep, SSD: short sleep duration (< 6h), CI: confidential interval

p-value was calculated by $\chi 2$ test.

Table S2. Odds ratio of NRS for alcohol and smoking among men with non-SSD

	Model 1 (N=23,077)			Model 2 (N=22,265)			Model 3 (N=19,950)		
	OR	95%CI	p-value	OR	95%CI	p-value	OR	95%CI	p-value
Alcohol /day (ref non-alcohol drink)									
≥0, <23g	0.91	0.80 - 1.03	0.128	0.96	0.84 - 1.10	0.579	0.93	0.80 - 1.07	0.292
≥23, <46g	0.96	0.82 - 1.12	0.611	1.05	0.89 - 1.23	0.594	0.97	0.82 - 1.16	0.767
≥46, <69g	1.18	0.95 - 1.46	0.142	1.22	0.96 - 1.54	0.098	1.09	0.85 - 1.40	0.485
≥69g	1.61	1.24 - 2.09	<0.001	1.51	1.14 - 2.01	0.004	1.39	1.03 - 1.86	0.031
Smoking (ref non-smoker)									
Light smoker	1.18	1.01 - 1.37	0.034	1.17	0.99 - 1.37	0.059	1.24	1.05 - 1.47	0.011
Heavy smoker	1.71	1.31 - 2.23	<0.001	1.43	1.08 - 1.89	0.013	1.52	1.13 - 2.05	0.006

Abbreviations: NRS: nonrestorative sleep, SSD: short sleep duration (< 6h), CI: confidential interval

Participants for whom data were missing were excluded from the analyses.

Model 1: Adjusted age group and interaction between alcohol and smoking.

Model 2: Model 1+ diet, exercise, sleep duration, and mental distress.

Model 3: Model 2+ marital status and education class.

p-value was calculated by the multiple logistic regression.

Table S3. Odds ratio of NRS for alcohol and smoking among women with non-SSD

	Model 1 (N=24,147)			Model 2 (N=23,184)			Model 3 (N=20,757)		
	OR	95%CI	p-value	OR	95%CI	p-value	OR	95%CI	p-value
Alcohol/day (ref non-alcohol drink)									
≥0, <23g	0.94	0.84 - 1.05	0.252	0.88	0.78 - 0.99	0.033	0.90	0.79 - 1.01	0.083
≥23, <46g	1.28	1.04 - 1.58	0.020	1.26	1.00 - 1.57	0.047	1.25	0.99 - 1.57	0.062
≥46g	1.57	1.15 - 2.12	0.004	1.48	1.06 - 2.06	0.021	1.31	0.92 - 1.87	0.132
Smoking (ref non-smoker)									
Light smoker	1.33	1.11 - 1.59	0.002	1.07	0.88 - 1.30	0.476	1.10	0.89 - 1.35	0.375
Heavy smoker	1.22	0.66 - 2.24	0.530	0.75	0.39 - 1.46	0.402	0.84	0.41 - 1.69	0.616

Abbreviations: NRS: nonrestorative sleep, SSD: short sleep duration (< 6h), CI: confidential interval

Participants for whom data were missing were excluded from the analyses.

Model 1: Adjusted age group and interaction between alcohol and smoking.

Model 2: Model 1+ diet, exercise, sleep duration, and mental distress.

Model 3: Model 2+ marital status and education class.

p-value was calculated by the multiple logistic regression.
